# Hitchhikers on floats to Arctic freshwater: Private aviation and recreation loss from aquatic invasion

**DOI:** 10.1007/s13280-019-01295-7

**Published:** 2019-12-04

**Authors:** Tobias Schwoerer, Joseph M. Little, Jennifer I. Schmidt, Kyle W. Borash

**Affiliations:** 1 Institute of Social and Economic Research, University of Alaska Anchorage, 3211 Providence Dr., Anchorage, AK 99508 USA; 2 International Arctic Research Center, University of Alaska Fairbanks, Fairbanks, AK 99775 USA; 3 School of Management, University of Alaska Fairbanks, 1731 South Chandalar Dr., Fairbanks, AK 99775 USA

**Keywords:** Alaska, Aquatic invasive species, Consumer welfare, Ecosystem service valuation, Ex-ante impact, Recreational aviation

## Abstract

This study of aviation-related recreation loss shows that a survey primarily aimed at collecting information on invasive species’ pathways can also be used to estimate changes in pathway-related ecosystem services. We present a case study for *Elodea* spp. (elodea), Alaska’s first known aquatic invasive plant, by combining respondents’ stated pre-invasion actual flights with stated post-invasion contingent behavior, plane operating costs, and site quality data. We asked pilots about the extent of continued flights should destinations become invaded and inhibit flight safety. We estimate a recreation demand model where the lost trip value to the average floatplane pilot whose destination is an elodea-invaded lake is US$185 (95 % CI $157, $211). Estimates of ecosystem damages incurred by private actors responsible for transmitting invaders can nudge actors to change behavior and inform adaptive ecosystem management. The policy and modeling implications of quantifying such damages and integration into more complex models are discussed.

## Introduction

Invasive species pose a threat to the health of aquatic ecosystems worldwide and affect ecosystem services that economic sectors such as recreation and fisheries depend upon (Rothlisberger et al. 2012). Since markets do not capture prices related to biological invasions, welfare measures of the damages remain largely unquantified (Finnoff et al. 2010). Non-market valuation can point to hidden costs but few studies have looked at aquatic invasive species (AIS) and those that have adopt ex-post perspectives measuring impact after the invasion had established (Rockwell 2003; Lovell et al. 2006; Marbuah et al. 2014). While such economic impact estimates can illuminate the damage already done, an ex-ante approach by contrast informs policy decisions and management actions to avoid damages, particularly if AIS pathways are well understood (Sepulveda et al. 2012). Ex-ante estimates are particularly important for AIS which are more difficult to detect compared to terrestrial invasive species and thus are more likely to be established before being detected increasing the cost of action. In such circumstances, ex-ante estimates provide the necessary data to weigh management costs against benefits of taking action.

Economic research on aquatic invasions often focuses on already established invasions with little attention to areas where the invasive species problem is in its infancy. For example, the invasion of *Dreissena* mussels in the Great Lakes, USA, has been known since the 1980s and economic research has focused on assessing economic impacts and pathways (Muirhead et al. 2009; Timar and Phaneuf 2009; Rothlisberger et al. 2012). In contrast, little research has looked at long-distance dispersal into remote regions of the world with largely intact ecosystems far from human development. The invasive species problem is just starting to become recognized in northern latitudes where it is a contributor to biodiversity loss (CAFF 2013; Schwörer et al. 2014). Ex-ante approaches can aid decision makers in selecting management options that minimize potential future damages and inform investments about the long-term economic benefits of preventing new arrivals and slowing the spread of existing invasions (Marbuah et al. 2014).

Quantifying the social values at stake informs the social–ecological assessments needed for adaptive ecosystem-based management and as such is an important contribution to evidence-based decision making (Folke 2006). Such decision making is especially challenging for aquatic systems where perturbations such as the introduction of AIS can cause regime shifts that trigger the loss of ecosystem services (Angeler et al. 2014). In such situations, resource managers are not only in need of information about the ecology of the waterbody at risk but also rely on data and cooperation from actors responsible for the ecosystem perturbations (Reyers et al. 2018). For example, quantifying predictive damages can nudge or incentivize actors to change their behavior (Bhargava and Loewenstein 2015) but also allow managers to weight cost of action against the avoided damages to resource users.

Especially for research related to biological invasions, past research has found strong bias towards investigation of ecological rather than social–ecological questions (Estévez et al. 2014). The combination of social and ecological information inform social–ecological models that aid in a structured decision-making process (Maguire 2004). An important characteristic of such models is that the net benefits associated with different management actions resulting in various outcomes are quantified (Polasky et al. 2011a, b). It is difficult to accurately and reliably measure expected net benefits, yet, they need to be known for testing new theories and improving the sustainability of complex social–ecological systems (Ostrom 2009). Also, integrating expected net benefits into structured decision making enhances risk communication and promotes trust between stakeholders and decision makers in areas of resource conflict which is often observed in the context of biological invasions (Estévez et al. 2014; Young et al. 2016).

The presented research was motivated by the recent discovery of Alaska’s first documented submersed freshwater aquatic invasive plant *Elodea* spp. (elodea). It was found in 2015 in Anchorage’s Lake Hood, the world’s busiest floatplane base (Hollander 2015). Known infestations are primarily in urban lakes and are being distributed by floatplanes to remote destinations across the state where the explosive and dense invasive plant growth creates safety hazards for pilots (Hollander 2014). In Lake Hood, the presence of dense aquatic vegetation has been a long-time safety concern for pilots requiring continued vegetation removal (CH2MHILL 2005). Also, dense aquatic plant growth such as observed with elodea can prevent pilots from accessing lakes for recreation. Since Alaska is mostly roadless, small single engine propeller planes with floats play a large role for commercial and private transportation during the summer (Gray 1980). The USA has the highest ownership of private planes per capita in the world with Alaska having 16 times as many aircraft per capita compared to other U.S. states and there are six times as many pilots (The Ninety-Nines 2016).

Very little is known about aviation-based recreation and in particular the risk of aviation-based AIS transmission (Carey et al. 2016). In order to inform managers and engage resource users about the potential net benefits of acting on AIS, this study had several research objectives. First, we wanted to quantify and show the aviation-based pathway for resource managers tasked with detecting new infestations. The second objective was to show floatplane pilots the hidden cost of their unintentional transmission of elodea to raise awareness and nudge them to change behavior that minimizes transmission risk. The third objective was to quantify important variables for later development of more complex social–ecological models that can aid in the further management of AIS by integrating social and economic with ecological data such as in quantitative risk and decision analysis (Maguire 2004; Verna et al. 2018).

We used an innovative approach combining spatial data elicited through an online survey with available site quality data to identify floatplane destinations and then estimate a recreation demand model. The approach extends previous exploratory research on the floatplane pathway (Carey et al. 2016) and borrows from the natural resource damage and recreation demand literature (Hausman et al. 1995). Resource damage and recreation demand approaches can apply random utility models (RUMs) to quantify non-market demand and associated welfare changes as a function of site quality (Shonkwiler and Shaw 2003; Scrogin et al. 2004; Landry et al. 2012). The approach has been applied to estimating changes in ecosystem services ex-post related to environmental disasters such as the Exxon Valdez and Deepwater Horizon oil spills (Carson et al. 2003; Glasgow and Train 2018).

We used the travel cost model in its traditional form which measures non-market values associated with existing recreation use (Trice and Wood 1958; Clawson 1959; Parsons 2017). We extended it to include a set of hypothetical questions where pilots were asked to state the number of two-way flights (trips) between home base and destinations. Destinations varied in environmental quality first assuming current pre-invasion conditions followed by hypothetical post-invasion conditions (Adamowicz et al. 1994; Englin and Cameron 1996). Most recent applications of the approach were used for environmental valuation of sport fishing experiences (Pokki et al. 2020) or to estimate the impacts of wind turbines on recreation (Kipperberg et al. 2019).

Our approach is also anchored in the larger literature on environmental impact assessment which over the past decade has seen a rise in stakeholder engagement and participatory approaches (Gray et al. 2017). In cases where quantitative social–ecological models require quantitative stakeholder input, structured survey techniques can provide consistent information and central tendencies related to environmental perceptions. These inputs can then be used to develop statistically robust models (Nelitz and Beardmore 2017). Lastly, our approach falls into citizen science where there is a need for improved data quality which we addressed through a structured elicitation technique (Dickinson et al. 2012).

Below we first describe the structured survey approach we used to elicit information on flight destinations and operating costs and lay out the econometric model, data compilation, and model estimation. Our study results indicate that elodea can cause significant lost trip value for recreational pilots. The article closes by discussing the merits of the approach and important policy implications.

## Materials and methods

### Survey

A stratified random sample of 1 015 floatplane-certified pilots residing in Alaska was drawn from the population of 2 625 pilots whose names, physical addresses, and certifications are published in the Airmen Certification Releasable Database (FAA 2015),^1^, ^2^ We divided the sample frame into an urban and rural strata following U.S. Census designations and oversampling the rural strata (U.S. Census Bureau 2010).

The survey was administered via Qualtrics Software between December 2015 and May 2016 (Qualtrics 2015). Pilots were first contacted using a letter of invitation including a URL address for completing the survey online and a US$2 incentive payment, followed by a post card reminder. The third contact included a reminder letter with hard copy of the survey and a stamped return envelope (Dillman 2007). Lastly, we called non-respondents for which phone numbers were available and digitized their response using the electronic survey.^3^

The web survey contained an awareness section about elodea, an electronic mapping tool that we programmed in JavaScript using Mapbox Outdoors general-purpose maps, and a section about plane operating costs and socio-demographics.

**Fig. 1:**
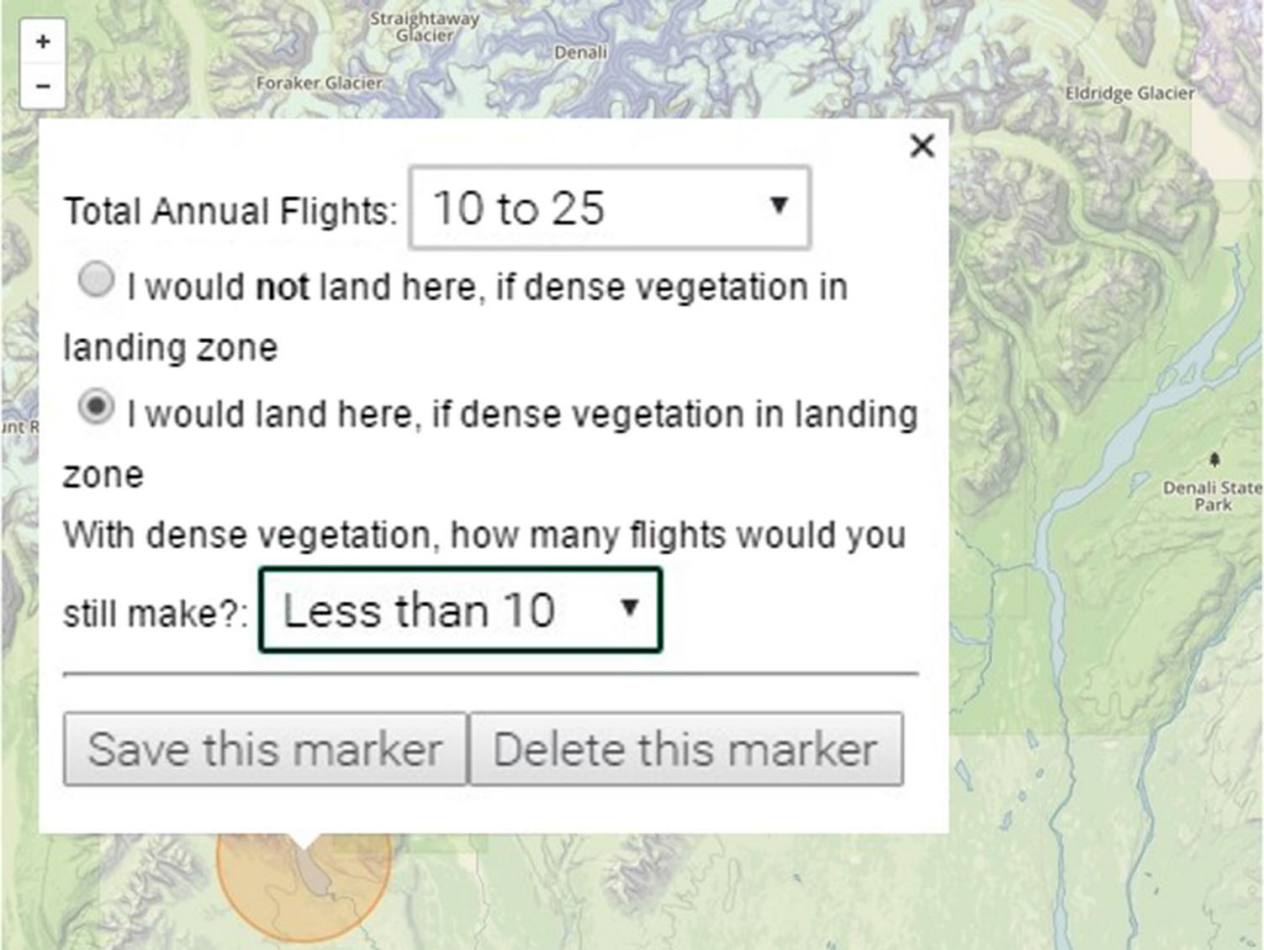
Computer screen view of online mapping tool for eliciting floatplane destinations

The mapping tool enabled precise identification of flying destinations while avoiding spatial ambiguity. The online-map was fixed to remain oriented North and allowed respondents to zoom without a maximum zoom level. Respondents were first asked about their home base followed by a request to mark their 2015 first-leg freshwater destinations (Fig. 1). Respondents placed an electronic marker onto a destination and a pop-up menu asked the pilot to state the 2015 annual flights to the marked destination and then select one of two statements: (1) I would **not** land here if dense vegetation in the landing zone, and (2) I would land here if dense vegetation in the landing zone. With dense vegetation, how many flights would you still make? (Fig. 1). Frequency of two-way flights between home base and destination was reported using the following intervals: < 10, 10–25, 25–50, 50–75, 75–100, and more than 100, where the midpoint of each interval was used for empirical analysis.

Ten key informant interviews helped refine the survey and flight frequency intervals, and justified focusing the mapping exercise on first-leg flights. Most pilots mentioned flying to a destination and then returned to their home base with few flights containing more than one destination. We defined this first-leg two-way flight pattern as a *flight trip* for further analysis.^4^ Assuming one flight trip per day, the selected flight trip intervals are consistent with season length (Rust’s Flying Service, pers. comm.).

Alternative approaches to data collection were not considered because they require more time to design, are more complex (e.g., discrete choice experiments), would place an additional burden on respondents reducing participation, or rely on the recording of recreation activity (Carson et al. 2009).

### Model

Below we describe the travel cost model that we used to estimate the change in ecosystem services given biological invasions. In principal, individual pilots spend varying amounts of money to access destinations (Parsons 2017). Destinations that are further away from the pilot’s home base are visited less and cost more to access and vice versa. Given the varying costs and frequencies to fly to destinations, we used the survey data to estimate the change in the average willingness to pay across pilots given a biological invasion to the pilot’s destination. We model a pilot’s decision to fly to a destination following random utility theory which allowed us to place pilots’ destination choices in the context of the set of available destinations. The underlying assumption of random utility theory is that pilots generally choose a destination they prefer over all other destinations and that this choice only in part can be explained by observation leaving some unexplained random error (McFadden 1973).

We think of the decision to fly as comprised of two parts, first selecting the destination followed by annual trips to that destination (Manski and McFadden 1981).^5^ This decision is reduced to just one level by using the number of flight trips as a frequency weight.^6^ The econometric specification is,

1 $$U_{nj} = V_{nj} + \varepsilon_{nj}$$

where *U*_*nj*_ is the overall utility of a destination alternative *j* to individual pilot *n* comprised of an observable *V*_*nj*_ and unobservable part of utility, *ε*_*nj*_. The underlying observable utility *V*_*nj*_ can be described in mathematical form as

2 $$V_{nj} = \gamma + \delta_{j} Z_{n} + \beta X_{nj}$$

where *γ* represented the average of all the unobserved sources of utility, *δ* a vector of coefficients measuring the contribution of *Z* a matrix of pilot-specific attributes, specifically here *Z*^A^ pilot age, *β* a vector of coefficients measuring the contribution of *X* a matrix of destination-specific attributes, specifically here *X*^E^ a dummy variable for hypothetical elodea-invaded destinations, *X*^C^ travel cost derived from plane-specific operating costs, and *X*^*S*^ and *X*^M^ reported hunting quality for sheep and moose, respectively.

Each individual pilot evaluated all destination alternatives, *U*_*j*_ for *j* = 1,…, *J* alternatives and chose the destination alternative with maximum utility, max(*U*_*j*_). The probability of an individual pilot choosing destination alternative *i,* was equal to the probability that the utility associated with alternative *i* was equal or greater than the utility of any other destination alternative, *U*_*j*_, in the choice set, thus *p*_*i*_ = p(*U*_*i*_ ≥ *U*_*j*_), where i ≠ *j* and *j* ∈ *j* = 1,…, *J*. Note, we use *i* instead of *j* to distinguish between the chosen destination alternative and all other destination alternatives.

We used Multinomial logit (MNL) and multinomial probit (MNP) to estimate the random utility models, commonly used for estimating recreation demand (McFadden 1973; Hausman et al. 1995; Chen et al. 1997). In the MNL, the pattern of substitution between destination alternatives is limited by the Independence of Irrelevant Alternatives (IIA) property. Under IIA, a change in one destination alternative has the same effect on all other destination alternatives. Thus, all destination alternatives are assumed to be equally dissimilar with none being more or less similar to each other (Hausman et al. 1995). As such, when the pilot chose destination alternative *i* from a set of *J* destination alternatives, the choice probability equaled:

3 $$p_{ni} = \frac{{e^{{\beta_{n} Z_{ni} }} }}{{\sum\limits_{j = 1}^{J} {e^{{\beta_{n} Z_{nj} }} } }}$$

where *i* ≠ *j* and *j* ∈ *j* = 1,…,*J* (McFadden 1973). In contrast, with the MNP, the probability ratio depends not only on the utility functions for alternatives *i* and *j* but all alternatives, thus relaxing the IIA assumption (Chen et al. 1997). The resulting choice probabilities are given by:

4 $$p_{ni} = \int_{ - \infty }^{\infty } {F_{j}\left( {\left( {X_{j} - X_{i} } \right)\beta_{n} + \varepsilon_{nj} } \right)d\varepsilon_{nj} }$$

where *F*_*j*_ is the joint distribution of the errors. Estimation of these choice probabilities relies on Monte Carlo simulation techniques such as Gibbs sampling.

The welfare changes estimated from either of the two recreation demand models are equal to the total derivative of the utility function (Eq. 2) with respect to changes in the elodea X^E^ and cost X^C^ attributes and expressed as follows (Hole 2007):

5 $$\frac{{dX^{C} }}{{dX^{E} }} = WTP_{{X^{E} }} =- \frac{{\beta_{ X^{E} }}}{{\beta_ {X^{C} }}}$$

Equation 5 represented the annual value lost per floatplane trip, a change in consumer welfare related to elodea invasion, *X*^E^. The same formula is used for deriving other welfare estimates related to sheep and moose hunting quality, where *X*^S^ and *X*^M^ replaces *X*^E^, respectively in Eq. 5. The introduction of elodea changes the vector of benefits pilots derive from a destination by altering accessibility, recreational quality, and other amenities. A measure of the benefits associated with these factors is equal to the difference between the pre- and post-invasion change in cost that keeps utility—the overall satisfaction of the pilot with the destination—unchanged. The loss in trip value can then be aggregated across the population of pilots to reflect the loss in consumer surplus, in other words, the loss in non-market value associated with potential invasions of floatplane destinations.

### Data compilation

**Fig. 2:**
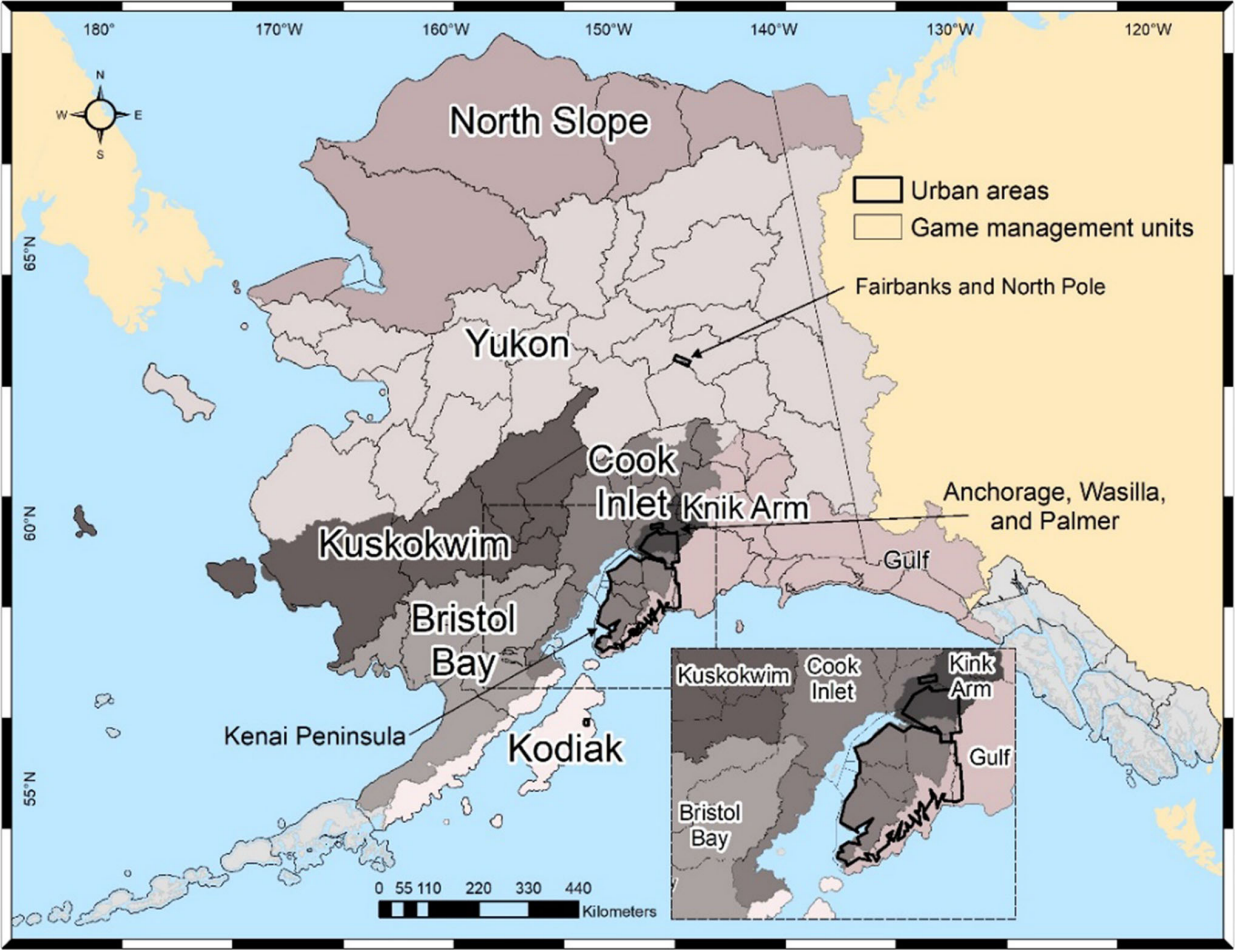
Eight regions defining destination alternatives in the recreation demand model (each shown in a different shade of gray), Game Management Units within these regions, and areas specified in the urban strata (see insert)

Since the survey did not ask about destination characteristics and the motivation of pilots, we relied on statewide publicly available site quality data. In order for substitution patterns to emerge and proper damage assessment to occur (Hausman et al. 1995), we created a panel dataset (Schwoerer et al. 2019). The pre-invasion actual flight information was combined with information on post-invasion contingent behavior as reported by pilots (Englin and Cameron 1996; Hynes and Greene 2013). Each respondent’s individual destinations were grouped into eight regions encompassing large watersheds defined by the National Hydrographic Dataset (NHD) (Fig. 2) (USGS 2017).

This aggregation was necessary for two reasons. First, the data showed more than 700 individual destinations, a number too large for estimation purposes. Many econometric software packages limit the number of alternatives in the choice model. Second, the regions closely align with watershed boundaries of smaller scale game management units (GMU) for which data on hunting quality is available (ADFG 2016).^7^ Hunting quality was assessed by calculating species-specific successful hunter ratios as reported within a GMU in 2015 for moose and sheep hunts (Table 1) (ADFG 2016). We calculated successful hunter ratios in order to capture both the recreation outcome and effort like catch per unit effort used in fisheries. It served as an indirect measure for the abundance of target species and thus as a site quality attribute (Skalski et al. 2005). If a GMU spanned multiple watersheds we used area differences to allocate hunters and harvest among watersheds. Consequently, if two pilots flew to the same watershed region but their individual destinations fell into different GMUs, hunting quality varied between the destinations.

**Table 1:** Successful hunter ratios by species and descriptive statistics on size and number of Game Management Units within each region, 2015

Region	Sheep Mean (SD)	Moose Mean (SD)
Gulf	0.16^a^	0.35 (0.27)
Knik Arm	0.15 (0.21)	0.16 (0.01)
Cook Inlet	0.18^a^	0.25 (0.10)
Kodiak	0.00^a^	0.31 (0.23)
Bristol Bay	0.00^a^	0.30 (0.09)
Kuskokwim	0.73^a^	0.57 (0.07)
North Slope	0.24 (0.27)	0.32 (0.31)
Yukon	0.20 (0.18)	0.36 (0.14)
All regions combined	0.22 (0.23)	0.34 (0.20)

Proportion of successful hunters varies by game management units within region. Not all regions contain sheep for harvest

^a^Regions with data limitations

It is recognized that hunting quality is only part of what sets one region apart from another and does not describe floatplane activity. Here, the variation in successful hunter ratios was solely used as a descriptor of how regions varied in harvest and wildlife viewing quality. Unlike other publicly available recreation-based data derived from angler and visitor surveys for example (Romberg 2014), the reported hunting data were more reliable for inclusion in the model. Unfortunately, additional data on other covariates were unavailable such as water depth, extent of aquatic vegetation, or water quality.

A no-fly alternative was included to account for the difference between pre- and post-invasion flight activity across all destination alternatives. The resulting panel data then contained two choice sets for each pilot (18 rows), where the first set of nine choice alternatives (regions) represented the 2015 flight pattern, and the second set showed hypothetical response under post-invasion conditions. Thus, we imply that destinations pre-invasion do not have dense vegetation but would have dense vegetation post-invasion. This assumption is justified as the mean native aquatic vegetation cover in un-invaded Alaska lakes is 27 %, whereas in invaded waters can reach 100 % (Rinella et al. 2008; Lane 2014).

A binary choice variable indicated to which region the pilot flew for both the pre-invasion and post-invasion choice sets. The remaining explanatory attributes included pilot age and travel cost. The cost to fly to each alternative region was individual-specific for regions the pilot chose to fly to and estimated for all regions including those the pilot did not choose to fly to. The stated floatplane operating cost, aviation fuel cost, pilot’s plane type and cruising speed were used to calculate a per km cost for each respondent multiplied by the weighted average of each respondent’s Euclidean distances between home base and destinations within region *j*. Costs associated with regions to which the pilot did not fly, were estimated using the pilot’s per km cost multiplied by the Euclidean distance between the pilot’s home base and centroid of the regions not chosen.

### Model estimation

Non-participation in the survey was assumed to be randomly distributed across the population of pilots and was addressed via weighting. Econometric analysis used frequency weights equal to the number of flight trips taken to respondents’ destinations in each region. The weight was further scaled to the population of pilots in each stratum, accounting for oversampling in rural areas and flights not taken. We estimated the models using the general linear model package in STATA with a logit and probit link, respectively (Hausman et al. 1995; StataCorp 2018).

White’s robust standard errors were used for inference as data collection possibly caused the explanatory attributes and the error term to not be identically distributed as assumed by the model (White 1980). For the damage assessment following Eq. 4, a 95 % confidence interval was estimated surrounding the mean using the Krinsky and Robb method with 2 000 replications (Krinsky and Robb 1986; Hole 2007).

## Results

### Survey response

Of the 1 015 initial mailings, 15 were undeliverable. A total of 444 pilots responded for a response rate of 44 %, which included 162 hard copy mail returns. The average web-based respondent took 24 min to complete the survey. A total of 239 pilots reported that they flew a floatplane in Alaska in 2015 and 229 of those provided mapping responses useful for analysis. Of the total respondents, 219 indicated not having flown in 2015, and four respondents did not answer whether they flew. Responses from rural areas were proportionally larger, likely due to oversampling in rural areas at the expense of under sampling in urban areas. Responses from other urban areas were proportional.

Half of the respondents were older than 58 years of age. Respondents’ median personal income before taxes in 2015 was US$135 000 compared to the most recent statewide median annual earnings of US$30 800 (Table 2) (U.S. Census Bureau 2017). Pilots varied most in the number of flight trips they took in 2015, on average between 30 and 40 flight trips over a roughly 100-day season. Table 2 presents additional respondent characteristics.

**Table 2:** Respondent characteristics

	Personal income^a^	2015 avg.# passengers	2015 flight trips^b^	Pilot age	Number of unique destinations	Max. flight distance (km)	Operating cost (US$/km)^c^
Mean	$137 786	1.41	36	58	4.23	257	$0.83
Median	$135 846	1.00	25	58	3	222	$0.75
Mode	$135 846	1.00	5	58	1	185	$0.78
SD	$70 101	1.13	46	11	5	162	$0.51
CV	0.51	0.80	1.28	0.19	1.18	0.63	0.61
Minimum	$25 000	0	5	26	1	3	$0.10
Maximum	$300 000	6.00	88	94	55	1 000	$2.97
Respondent count	157	213	229	183	229	211	173

^a^Before taxes

^b^Respondents reported the number of trips using intervals from which the midpoint was taken for further analysis

^c^Estimated based on cruising speed of plane type and stated operating cost. Varies by respondent and aircraft type

The annual average number of unique destinations to which pilots flew from their home base was between four and five, a limited number of destinations (Table 2). This result likely suggests that familiarity with local conditions is important to pilots flying in Alaska. Consequently, there is also a limited number of substitute destinations to which pilots prefer to fly. Only one pilot indicated to have increased flights to one destination, with no change to another, and decreasing or stopping flights to five remaining destinations. This result supports our focus on existing pre-invasion landing destinations rather than new substitute destinations.

We did not conduct a non-response survey to address specific selection bias. However, using a t-test and the most recent American Community Survey’s 5-year estimates of median household income and per capita income, we showed that there are no statistically significant income differences between Census-designated places with non-respondents and Census-designated places with respondents (*t*-test, *p* = 0.0008 and *p* = 0.004, respectively) (U.S. Census Bureau 2017). Thus, the characteristics of the sample and the t-test suggest that based on income—an important contributor to whether pilots are able to fly or not—non-respondents are likely similar to respondents.

Half of the respondents stated that they would no longer have flown to destinations they flew to in 2015 if dense aquatic vegetation would have been in the landing zone (Table 3). About 75 % of respondents had heard about elodea and reported safety concerns flying to destinations that were shallow and already required caution for landings and take-offs. In follow-up phone interviews, pilots identified destinations by talking about individual lake characteristics such as water depth and terrain features. For example, some pilots considered continuing to land in destinations with larger water depth because elodea invasions would predominately occur in shallower parts of a lake or waterbody. Pilots also mentioned that they would have reduced or eliminated flying to destinations with shallower water depth as these locations are more prone to elodea infestations, and increase flying to deeper lakes with less hazardous conditions.^8^ Consequently, contingent post-invasion flying behavior could lead to a downward shift in trip demand for some destinations while it could lead to an upward shift in other destinations (Table 3). We estimated that elodea invasions would reduce the total statewide number of flight trips by two thirds, assuming no site substitution.

**Table 3:** Recreational pilots’ stated change in flight behavior due to invasion, *n* = 229

	Continue flying				Stop flying
	To all their destinations			Only to some destinations with flight trip reductions	
	Flight trip increases to some destinations^a^	No change	Flight trip reductions to some destinations		
Pilot count (%)	4 (2 %)	39 (17 %)	36 (16 %)	35 (15 %)	115 (50 %)
Mean % change in annual flight trips	+ 120 %	0 %	− 40 %	− 58 %	− 100 %

^a^Flight trip increases to some destinations are due to flight trip decreases in other destinations suggesting some degree of substitution

### Empirical results

We used maximum likelihood optimization to fit a Multinomial logit (MNL) and multinomial probit (MNP) model. The signs were as expected for the estimated coefficients explaining the choice of destination alternative. All predictor variables were statistically significant with *p* values less than alpha set at 0.05. The negative coefficients for the elodea invasion and cost variables allowed for the calculation of lost flight trip value (Table 4). This model result indicated that destinations infested with elodea are more expensive to travel to and are avoided while lakes with hunting opportunities are preferred (Table 4). This empirical result was supported by more than three quarters of respondents indicating that prior to the survey they had heard about the spread of elodea and were aware of the floatplane safety risk it poses. Not surprising were the coefficients for hunting quality, considering that Alaska has the highest participation rate in wildlife-related recreation by state residents among U.S. states (U.S. Fish and Wildlife Service and U.S. Census Bureau 2013). The positive coefficient on the age variable was expected and reflects that flying is an expensive hobby reserved for those with time and sufficient disposable income to pursue the activity. The income variable was not included in the model due to correlation with trip cost and trip frequency.

**Table 4:** Estimated coefficients explaining choice of destination alternative and estimated change in consumer welfare per flight trip

Coefficient	MNL			MNP		
	Mean (robust SE)	95 % Confidence interval		Mean (robust SE)	95 % Confidence interval	
Elodea invasion	− 0.296 (0.02)*	− 0.337	− 0.256	− 0.183 (0.01)*	− 0.206	− 0.159
Cost	− 0.002 (0.00)*	− 0.002	− 0.002	− 0.001 (0.00)*	− 0.001	− 0.001
Moose hunting quality	1.431 (0.13)*	1.183	1.679	0.836 (0.07)*	0.695	0.977
Sheep hunting quality	2.270 (0.08)*	2.117	2.424	1.279 (0.05)*	1.190	1.369
Age	0.010 (0.00)*	0.009	0.011	0.006 (0.00)*	0.005	0.007
Constant	0.398 (0.05)*	0.306	0.490	0.266 (0.03)*	0.211	0.321
AIC (deviation)	1.085			1.085		
BIC	− 1 018 526			− 1 018 552		
Log ps likelihood	− 53 109			− 53 096		
Welfare change						
Elodea invasion	− $178	− $205	− $151	− $185	− $157	− $211
Moose hunting quality	$861	$736	$981	$848	$726	$965
Sheep hunting quality	$1 366	$1 215	$1 531	$1 298	$1 162	$1 447

*Coefficients are statistically significant as their *p* values are less than alpha set at 0.05

The coefficients for the MNL and the MNP models were comparable in sign and magnitude with similar high precision, yet the MNP offered better fit compared to the MNL as shown by the smaller Bayesian information criterion (BIC) value. The mean lost flight trip value estimated by the MNL was − $178 (95 % CI − $205, − $151) and by the MNP equaled − $185 (95 % CI − $211, − $157).

The similarity among model parameters and WTP may suggest that the IIA assumption had little consequence as long as sufficient data quality minimized the amount of unobserved heterogeneity (Hensher et al. 2005). Additionally, a null model was estimated for both MNL and MNP. In both cases the AIC was equal to 1.14 suggesting that inclusion of the covariates results in a better model.

## Discussion

Invasive species management is one example where resource managers often face decisions requiring rapid response to avoid ecosystem damages but lack adequate information to support their decisions (Liu et al. 2012). In this study, we showed that combining data on stated recreation site visits contingent on the presence of an invasive species with data on site quality can be used to estimate potential invasion-driven changes in cultural ecosystem services through diminished recreation access. In other words, we estimated the potential change in non-market value that aviation-based recreationists would lose given a biological invasion occurred in a preferred recreation site. Such empirical evidence can feed into structured decision-making models for managers to weigh the cost of response with its quantified benefits—the avoided loss to recreationists (Liu et al. 2012; Estévez et al. 2014; Young et al. 2016).

Our contributions are twofold. We filled an important knowledge gap about aviation-based long-range transmission of AIS and associated recreation-based ecosystem service loss. Key data gaps remain related to incentivizing and changing human behavior and quantifying ecosystem service impacts (Epanchin-Niell 2017). We illuminated hidden non-market economic impacts accruing to actors responsible for AIS spread enabling targeted nudges and incentives for those actors to change their behavior and reduce transmission risk (Bhargava and Loewenstein 2015). Our study also contributes to the monetary value-domains that are increasingly important for decision making based on more complex social–ecological systems analysis (Martín-López et al. 2014). Quantifying potential changes in ecosystem services accruing directly to stakeholders feeds into quantitative risk and decision models that are increasingly part of structured decision making in resource management (Suedel et al. 2007; Gregory and Long 2009; Liu et al. 2012).

Advantages of the modeling approach, beyond the combination of actual pre- and contingent post-invasion behavior, are centered upon integrating existing place-specific data to describe how the destinations vary. The integration of such stated and revealed preference data avoids potential biased welfare estimates that are a concern in stated preference techniques (Crastes dit Sourd et al. 2018). Our approach also reduces the response burden by eliminating additional survey questions that would be necessary to directly link motivational decision variables to destination choice. Since our approach does not establish this link, location-specific data quality are important and the reason why we relied on reported hunting success.

The inclusion of hunting quality could explain inelastic trip demand for destinations where hunting quality was high and floatplane access limited as often is the case for sheep hunting (Miller and McCollum 1994). In other words, the pilot would have continued to fly to the destination despite an elodea invasion and may have been willing to take more risk during landing or take-off in order to pursue what they perceive as a high-quality hunt. While successful hunter ratios are a good indicator of hunting and wildlife viewing quality and one potentially motivating factor for flying, there are unknown motivational drivers such as solitude or flightseeing the model did not capture.

Due to the very limited literature on aviation-based recreation (Carey et al. 2016) and lack of economic valuation for personal aviation-based recreation, the study’s welfare estimates cannot be directly compared to studies similar in scope and geography. However, since we included site quality variables related to hunting quality, we were able to validate the model’s welfare changes for hunting quality (Table 4). Consumer surplus values have recently been estimated for hunting and wildlife viewing in Alaska where per trip mean estimates ranged between US$438 (US$268) per resident hunter (viewer) and US$765 (USD$858) per visiting hunter (viewer) (Buckley 2014). This study’s higher estimates of US$861 for moose and $1366 for sheep hunting quality are comparable considering that floatplanes are the most expensive transportation mode and the previous study measured per-person values. Considering an average of one to two passengers per flight trip (Table 2), previous studies confirm our hunting-related welfare estimates and validate our model (Table 4).

The welfare losses estimated here are at best lower bounds to the actual economic losses and do not account for the wider more complex interactions within social–ecological systems that influence human wellbeing (Reyers et al. 2013). First, the study’s focus on recreational pilots leaves out potential production loss in the commercial sector. Second, the preferences and economic values of passengers were not considered. Third, this analysis concentrates on travel cost using operating cost as a proxy, ignoring pilots’ opportunity cost of time, even though one could argue that recreation has little to do with labor supply decisions. Fourth, the estimates do not capture ecosystem services provided by the water bodies and influenced by potential elodea infestations, such as sport fishing, hiking, hunting, and other local amenities that depend on viable floatplane access. Finally, non-use values may be held by society and future generations for waterbodies with ecological and cultural significance, thus existence and bequest values are not included.

The fielded survey was not designed to capture the full suite of contingent behavior reflected in all substitution sites. The reason for this approach was simplicity and a focus on destination information with high data quality that kept attrition to a minimum. Despite this drawback, we were able to account for substitution between landing destinations each pilot was familiar with. Even though the survey instrument did not specifically ask for a second-best destination, assuming the pilot’s existing landing destination becomes invaded, the approach was able to estimate the change in trip demand among the pilot’s existing set of destinations.^9^

The study also finds that the average Alaska floatplane pilot flies to fewer than five destinations, which suggests that pilots prefer a limited number of locations. More than three quarters of all surveyed pilots would either stop flying or reduce flights to destinations that have dense vegetation in the landing zone. Risk aversion may reduce site substitution behavior since exploring unknown destinations presents a risk for pilots not familiar with water depth and other localized conditions important to flight safety. Alaska’s very remote landscape and often severe weather may also play a role. Therefore, the pattern of substitution favors each pilot’s existing (pre-invasion) set of locations. This fact helps to underscore why the survey focused on collecting data on preferred destinations over hypothetical alternates. Avoiding questions about hypothetical alternate destinations may have also helped to reduce the potential for hypothetical bias. With the data at hand, however, there is no way to test for this possibility but it is one aspect where the research could be expanded.

The geographic scale of Alaska along with the large number of identified floatplane destinations introduces data complexities that are more readily addressed by the data collection and modeling approach presented such as those that combine stated and revealed preference data (von Haefen and Phaneuf 2008; Abildtrup et al. 2015). Specifically, a nested model would have served as a good alternative addressing a complex decision process related to destination choice. While a nesting structure would have relaxed the IIA property and allowed for the estimation of region-specific inclusive values, the demands on data quality are higher. In addition, the nested model could fail to be implemented as it requires inclusive value coefficients to be smaller than one, often necessitating re-specification of the nesting structure which does not always guarantee successful estimation (Hausman et al. 1995).

Poisson or negative binomial specifications are alternative distributional assumptions that could be made in this instance. Even though these models are used for estimating recreation demand, their application to this damage assessment is limited as their distributional assumptions are often violated resulting in biased welfare estimates. Therefore they were not considered for this study (Blaine et al. 2015). Lastly, an alternative-specific conditional logit model was specified but was not implemented due to poor fit (McFadden 1973).^10^

## Conclusions

We demonstrated that a survey primarily aimed at collecting information on invasive species’ pathways can also be used to estimate changes in pathway-related ecosystem services. We used an innovative approach to ecosystem service valuation that combined spatial data elicited through an online survey with available site quality data to estimate a recreation demand model. The approach is not only applicable for informing social–ecological models related to AIS management but can be used to elicit resource users more broadly about their change in use patterns and associated change in ecosystem services related to expected future environmental change. As such, the approach informs adaptive management by illuminating potential loss in ecosystem services that managers can account for when pre-emptively managing ecosystems to minimize long-term risk to resource users.

## Footnotes

1 According to the FAA, opt-out rates for not wanting to release personal data in this public database are < 1%.

2 We excluded Southeast Alaska because floatplane bases are almost exclusively in saltwater, minimizing freshwater AIS risk, AIS have not been found there, and only 8% of floatplane pilots reside there.

3 Phone numbers were purchased from a private marketing company.

4 The survey results presented thereafter underline the fact that Alaska floatplane pilots do not fly to many different destinations limiting the frequency of multi-destination flights. For this and for reasons of keeping the data collection and analysis simple we did not investigate multi-destination flights.

5 One could argue that there is a third level—flight distance. Respondents indicated small sets of destinations with a mean of four and median of three destinations (Table 2).

6 Frequency weights indicate duplicate observations and are integers. If the frequency weight for an observation is equal to three that means that there are three identical observations.

7 The Alaska Department of Fish and Game divides Alaska into game management units (GMU) which roughly align with watershed boundaries consistent with each region *j*.

8 In addition, weather conditions, pilot skills, and plane models are significant drivers determining access which were not incorporated into the model for reasons discussed earlier.

9 Also, the aggregation of destinations into regions for model estimation limits substitutability options.

10 The aggregated data and small sample did perhaps not offer enough variance to estimate coefficients specific to each region.
